# Supplementary material for: Pyruvate Dehydrogenase Kinase 1 inhibition mediated oxidative phosphorylation enhancement in cartilage promotes osteoarthritis progression
Source: BMC Musculoskelet Disord. 2023 Jul 20;24:597. doi: 10.1186/s12891-023-06585-6 (PMC10357736; doi:10.1186/s12891-023-06585-6)
Supplement: Supplementary file 1 — Additional file 1. [file 12891_2023_6585_MOESM1_ESM.zip › KEGG.Gsea.1653622432464/KEGG_VIBRIO_CHOLERAE_INFECTION.html]

Details for gene set KEGG\_VIBRIO\_CHOLERAE\_INFECTION[GSEA]

|  || Dataset | 55457\_collapsed\_to\_symbols.cls.cls #OA\_versus\_normal.cls.cls #OA\_versus\_normal\_repos |
| Phenotype | cls.cls#OA\_versus\_normal\_repos |
| Upregulated in class | OA |
| GeneSet | KEGG\_VIBRIO\_CHOLERAE\_INFECTION |
| Enrichment Score (ES) | 0.388903 |
| Normalized Enrichment Score (NES) | 1.6014153 |
| Nominal p-value | 0.013477089 |
| FDR q-value | 0.04026776 |
| FWER p-Value | 0.534 |
Table: GSEA Results Summary

  

Fig 1: Enrichment plot: KEGG\_VIBRIO\_CHOLERAE\_INFECTION      
 Profile of the Running ES Score & Positions of GeneSet Members on the Rank Ordered List

  

| SYMBOL | TITLE | RANK IN GENE LIST | RANK METRIC SCORE | RUNNING ES | CORE ENRICHMENT || 1 | ATP6V1C1 | ATPase H+ transporting V1 subunit C1 [Source:HGNC Symbol;Acc:HGNC:856] | 17 | 1.125 | 0.0552 | Yes |
| 2 | KDELR3 | KDEL endoplasmic reticulum protein retention receptor 3 [Source:HGNC Symbol;Acc:HGNC:6306] | 77 | 0.950 | 0.0983 | Yes |
| 3 | ATP6V0D1 | ATPase H+ transporting V0 subunit d1 [Source:HGNC Symbol;Acc:HGNC:13724] | 135 | 0.872 | 0.1377 | Yes |
| 4 | ATP6V1E1 | ATPase H+ transporting V1 subunit E1 [Source:HGNC Symbol;Acc:HGNC:857] | 287 | 0.758 | 0.1640 | Yes |
| 5 | ATP6V0B | ATPase H+ transporting V0 subunit b [Source:HGNC Symbol;Acc:HGNC:861] | 382 | 0.713 | 0.1926 | Yes |
| 6 | KCNQ1 | potassium voltage-gated channel subfamily Q member 1 [Source:HGNC Symbol;Acc:HGNC:6294] | 391 | 0.709 | 0.2276 | Yes |
| 7 | ATP6V1G1 | ATPase H+ transporting V1 subunit G1 [Source:HGNC Symbol;Acc:HGNC:864] | 515 | 0.655 | 0.2509 | Yes |
| 8 | ATP6V0E2 | ATPase H+ transporting V0 subunit e2 [Source:HGNC Symbol;Acc:HGNC:21723] | 532 | 0.647 | 0.2821 | Yes |
| 9 | PLCG2 | phospholipase C gamma 2 [Source:HGNC Symbol;Acc:HGNC:9066] | 725 | 0.588 | 0.2967 | Yes |
| 10 | ATP6V1D | ATPase H+ transporting V1 subunit D [Source:HGNC Symbol;Acc:HGNC:13527] | 750 | 0.581 | 0.3241 | Yes |
| 11 | PRKACB | protein kinase cAMP-activated catalytic subunit beta [Source:HGNC Symbol;Acc:HGNC:9381] | 771 | 0.576 | 0.3514 | Yes |
| 12 | SEC61G | SEC61 translocon subunit gamma [Source:HGNC Symbol;Acc:HGNC:18277] | 1163 | 0.485 | 0.3453 | Yes |
| 13 | ATP6V0E1 | ATPase H+ transporting V0 subunit e1 [Source:HGNC Symbol;Acc:HGNC:863] | 1207 | 0.474 | 0.3658 | Yes |
| 14 | ATP6V1H | ATPase H+ transporting V1 subunit H [Source:HGNC Symbol;Acc:HGNC:18303] | 1304 | 0.457 | 0.3813 | Yes |
| 15 | KDELR2 | KDEL endoplasmic reticulum protein retention receptor 2 [Source:HGNC Symbol;Acc:HGNC:6305] | 1486 | 0.431 | 0.3889 | Yes |
| 16 | ATP6AP1 | ATPase H+ transporting accessory protein 1 [Source:HGNC Symbol;Acc:HGNC:868] | 1866 | 0.377 | 0.3783 | No |
| 17 | KDELR1 | KDEL endoplasmic reticulum protein retention receptor 1 [Source:HGNC Symbol;Acc:HGNC:6304] | 2227 | 0.336 | 0.3672 | No |
| 18 | ATP6V0C | ATPase H+ transporting V0 subunit c [Source:HGNC Symbol;Acc:HGNC:855] | 2790 | 0.271 | 0.3371 | No |
| 19 | MUC2 | "mucin 2, oligomeric mucus/gel-forming [Source:HGNC Symbol;Acc:HGNC:7512]" | 2978 | 0.252 | 0.3352 | No |
| 20 | ERO1A | endoplasmic reticulum oxidoreductase 1 alpha [Source:HGNC Symbol;Acc:HGNC:13280] | 3686 | 0.187 | 0.2895 | No |
| 21 | ATP6V1G2 | ATPase H+ transporting V1 subunit G2 [Source:HGNC Symbol;Acc:HGNC:862] | 3709 | 0.184 | 0.2971 | No |
| 22 | PRKCA | protein kinase C alpha [Source:HGNC Symbol;Acc:HGNC:9393] | 3728 | 0.183 | 0.3049 | No |
| 23 | PDIA4 | protein disulfide isomerase family A member 4 [Source:HGNC Symbol;Acc:HGNC:30167] | 3756 | 0.181 | 0.3119 | No |
| 24 | ATP6V1F | ATPase H+ transporting V1 subunit F [Source:HGNC Symbol;Acc:HGNC:16832] | 3833 | 0.173 | 0.3146 | No |
| 25 | ATP6V0A1 | ATPase H+ transporting V0 subunit a1 [Source:HGNC Symbol;Acc:HGNC:865] | 4371 | 0.123 | 0.2790 | No |
| 26 | ADCY9 | adenylate cyclase 9 [Source:HGNC Symbol;Acc:HGNC:240] | 5191 | 0.057 | 0.2182 | No |
| 27 | SEC61A1 | SEC61 translocon subunit alpha 1 [Source:HGNC Symbol;Acc:HGNC:18276] | 5378 | 0.041 | 0.2058 | No |
| 28 | ATP6V0A4 | ATPase H+ transporting V0 subunit a4 [Source:HGNC Symbol;Acc:HGNC:866] | 5968 | -0.002 | 0.1601 | No |
| 29 | PRKCB | protein kinase C beta [Source:HGNC Symbol;Acc:HGNC:9395] | 6453 | -0.039 | 0.1243 | No |
| 30 | ATP6V1B2 | ATPase H+ transporting V1 subunit B2 [Source:HGNC Symbol;Acc:HGNC:854] | 7074 | -0.086 | 0.0804 | No |
| 31 | ARF1 | ADP ribosylation factor 1 [Source:HGNC Symbol;Acc:HGNC:652] | 7152 | -0.092 | 0.0791 | No |
| 32 | TCIRG1 | "T cell immune regulator 1, ATPase H+ transporting V0 subunit a3 [Source:HGNC Symbol;Acc:HGNC:11647]" | 7600 | -0.127 | 0.0507 | No |
| 33 | ATP6V1A | ATPase H+ transporting V1 subunit A [Source:HGNC Symbol;Acc:HGNC:851] | 8013 | -0.156 | 0.0264 | No |
| 34 | SEC61B | SEC61 translocon subunit beta [Source:HGNC Symbol;Acc:HGNC:16993] | 8510 | -0.194 | -0.0024 | No |
| 35 | TJP1 | tight junction protein 1 [Source:HGNC Symbol;Acc:HGNC:11827] | 8523 | -0.195 | 0.0065 | No |
| 36 | ADCY3 | adenylate cyclase 3 [Source:HGNC Symbol;Acc:HGNC:234] | 8572 | -0.199 | 0.0128 | No |
| 37 | PRKCG | protein kinase C gamma [Source:HGNC Symbol;Acc:HGNC:9402] | 8582 | -0.200 | 0.0221 | No |
| 38 | ATP6V0A2 | ATPase H+ transporting V0 subunit a2 [Source:HGNC Symbol;Acc:HGNC:18481] | 8843 | -0.221 | 0.0130 | No |
| 39 | GNAS | GNAS complex locus [Source:HGNC Symbol;Acc:HGNC:4392] | 8929 | -0.226 | 0.0177 | No |
| 40 | TJP2 | tight junction protein 2 [Source:HGNC Symbol;Acc:HGNC:11828] | 9772 | -0.298 | -0.0328 | No |
| 41 | PRKACG | protein kinase cAMP-activated catalytic subunit gamma [Source:HGNC Symbol;Acc:HGNC:9382] | 10466 | -0.370 | -0.0682 | No |
| 42 | PRKACA | protein kinase cAMP-activated catalytic subunit alpha [Source:HGNC Symbol;Acc:HGNC:9380] | 10611 | -0.384 | -0.0601 | No |
| 43 | PLCG1 | phospholipase C gamma 1 [Source:HGNC Symbol;Acc:HGNC:9065] | 10722 | -0.395 | -0.0488 | No |
| 44 | CFTR | CF transmembrane conductance regulator [Source:HGNC Symbol;Acc:HGNC:1884] | 10920 | -0.420 | -0.0430 | No |
| 45 | SEC61A2 | SEC61 translocon subunit alpha 2 [Source:HGNC Symbol;Acc:HGNC:17702] | 11352 | -0.479 | -0.0525 | No |
| 46 | PRKX | protein kinase X-linked [Source:HGNC Symbol;Acc:HGNC:9441] | 11634 | -0.522 | -0.0481 | No |
| 47 | SLC12A2 | solute carrier family 12 member 2 [Source:HGNC Symbol;Acc:HGNC:10911] | 11744 | -0.540 | -0.0295 | No |
| 48 | ATP6V1B1 | ATPase H+ transporting V1 subunit B1 [Source:HGNC Symbol;Acc:HGNC:853] | 12206 | -0.646 | -0.0329 | No |
| 49 | ACTG1 | actin gamma 1 [Source:HGNC Symbol;Acc:HGNC:144] | 12613 | -0.806 | -0.0240 | No |
| 50 | ACTB | actin beta [Source:HGNC Symbol;Acc:HGNC:132] | 12757 | -0.920 | 0.0111 | No |
Table: GSEA details [plain text format]

  

Fig 2: KEGG\_VIBRIO\_CHOLERAE\_INFECTION      
 Blue-Pink O' Gram in the Space of the Analyzed GeneSet

  

Fig 3: KEGG\_VIBRIO\_CHOLERAE\_INFECTION: Random ES distribution      
 Gene set null distribution of ES for **KEGG\_VIBRIO\_CHOLERAE\_INFECTION**
